# *Moringa oleifera* Lam. Seed Lectin (WSMoL) Attenuates Parkinsonian Symptoms in Mice via Anti‐Inflammatory, Antioxidant, and Neurotransmitter‐Modulating Effects

**DOI:** 10.1002/cbdv.71646

**Published:** 2026-08-31

**Authors:** Leydianne Leite de Siqueira Patriota, Emanuel Davi Lima de Matos Leão, Maria Nívea Bezerra da Silva, Amanda de Oliveira Marinho, Bárbara Raíssa Ferreira de Lima, Jainaldo Alves da Costa, Luciclaudio Cassimiro de Amorim, Valéria Bianca de Souza Santos, Patrícia Maria Guedes Paiva, Michelly Cristiny Pereira, Daniella Carla Napoleão, Jorge Vinícius Fernandes Lima Cavalcanti, Leucio Duarte Vieira, Michelle Melgarejo da Rosa, Thiago Henrique Napoleão

**Affiliations:** ^1^ Departamento de Bioquímica, Centro de Biociências Universidade Federal de Pernambuco Recife Brazil; ^2^ Departamento de Fisiologia e Farmacologia, Centro de Biociências Universidade Federal de Pernambuco Recife Brazil; ^3^ Departamento de Engenharia Química, Centro de Tecnologia e Geociências Universidade Federal de Pernambuco Recife Brazil; ^4^ Núcleo de Pesquisa em Inovação Terapêutica Suely Galdino (NUPIT) Universidade Federal de Pernambuco Recife Brazil

**Keywords:** lectin, neuroinflammation, Parkinson's disease, rotenone

## Abstract

This work investigated the effects of *Moringa oleifera* seed lectin (WSMoL) on motor performance, neuroinflammation, and oxidative stress in a rotenone‐induced model of Parkinson's disease (PD). Male mice were daily administered rotenone (3 mg/kg, i.p.) 1 h after being treated with WSMoL (1, 2, or 4 mg/kg, i.p.), levodopa (L‐DOPA, 10 mg/kg), or vehicle for 10 days. To assess dopaminergic involvement, another group received the D1 receptor antagonist SCH‐23390 co‐administered with WSMoL (4 mg/kg). The healthy control group received vehicle only. Motor performance was evaluated using the rotarod, cylinder, and open field tests. Brain levels of monoamines and cytokines as well as redox status were measured. WSMoL prevented the rotenone‐related deficits in motor performance, which was accompanied by non‐depleted dopamine and serotonin levels. WSMoL also mitigated the increase of IL‐6, IL‐17A, IFN‐γ, and TNF‐α. In addition, it prevented oxidative stress by avoiding the exacerbation of superoxide production, lipid peroxidation, and NADPH oxidase activity, while maintaining catalase, superoxide dismutase (SOD), and reduced glutathione (GSH) levels. The data show that D1 receptor‐mediated signaling contributes to WSMoL effects. In conclusion, WSMoL attenuates motor deficits and neuroinflammation in a PD model, which supports its potential as a neuroprotective agent and warrants further mechanistic and translational studies.

## Graphical abstract

WSMoL attenuates motor impairment, neuroinflammation, oxidative stress, and monoamine depletion in a rotenone‐induced model of Parkinson's disease. The involvement of D1 receptor‐mediated signaling is demonstrated, providing new insights into the mechanisms underlying its neuroprotective effects and supporting WSMoL as a promising candidate for further therapeutic investigation.

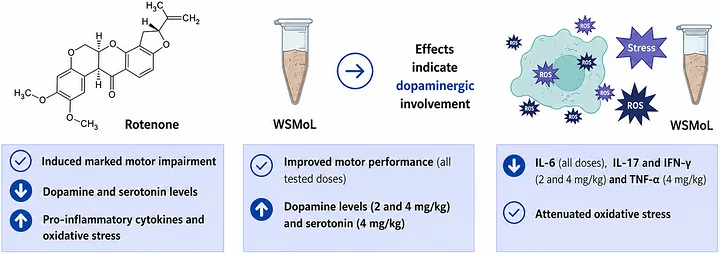

## 1 Introduction

Parkinson's disease (PD) is the second most common progressive neurodegenerative disorder, affecting both the central and peripheral nervous systems [1, 2, 3]. Currently, more than six million people worldwide live with PD, with approximately 2% of affected individuals being over 65 years old [2, 4, 5]. As global life expectancy rises, the number of cases is expected to reach nine million by 2030, placing increasing pressure on economies and healthcare systems [6, 7]. This escalating burden highlights the urgent need for new disease‐modifying therapies.

The hallmark features of PD include extensive neuroinflammation, elevated oxidative stress, progressive loss of dopaminergic neurons, particularly in the substantia nigra, and the accumulation of α‐synuclein [2, 8]. Dopamine deficiency underlies the core motor symptoms, tremor, bradykinesia, rigidity, and postural instability [9], while PD concurrently disrupts other neurotransmitter systems, including serotonergic, noradrenergic, and cholinergic pathways [6]. As a result, patients often experience non‐motor symptoms, such as cognitive impairment, mood disorders, and autonomic dysfunction [10]. Current treatments for PD rely heavily on dopamine replacement therapy, including levodopa (a dopamine precursor) and dopamine agonists. While these approaches alleviate symptoms, they are often associated with long‐term complications such as motor fluctuations, hallucinations, and cognitive decline [11, 12].

Mitochondrial dysfunction, particularly complex I inhibition, has emerged as a central mechanism in PD pathogenesis, contributing to oxidative stress, synaptic loss, and neuronal death [13, 14]. To mimic this pathology, the pesticide rotenone is widely used in experimental studies. Rotenone inhibits mitochondrial complex I, reducing ATP production and increasing reactive oxygen species, thereby selectively impairing dopaminergic neurons, inducing α‐synuclein aggregation, and disrupting cytoskeletal integrity. This closely recapitulates key aspects of PD [15].

Natural compounds with antioxidant and anti‐inflammatory properties have gained attention as potential neuroprotective agents [16, 17]. Among these, lectins (carbohydrate‐binding proteins) have demonstrated therapeutic promise by modulating neuroinflammation and neuroplasticity [18]. One such lectin, WSMoL, derived from *M. oleifera* Lam. seeds, has exhibited anxiolytic‐ and antidepressant‐like effects in acute neurobehavioral models of anxiety and depression in mice. These effects appear to be associated with modulation of serotonergic (5‐HT2A/2C receptors), adrenergic (α2‐adrenoceptors), and dopaminergic (D1 receptors) systems and also depend on the lectin's carbohydrate recognition domain [19, 20]. In addition, WSMoL treatment alleviated anxiety‐ and depression‐like behaviors in animals subjected to four weeks of chronic stress. This effect was accompanied by decreased levels of circulating pro‐inflammatory markers and attenuation of neuroinflammation in the brain. In this model, WSMoL significantly increased levels of dopamine, serotonin, and noradrenaline [21].

**FIGURE 1:**
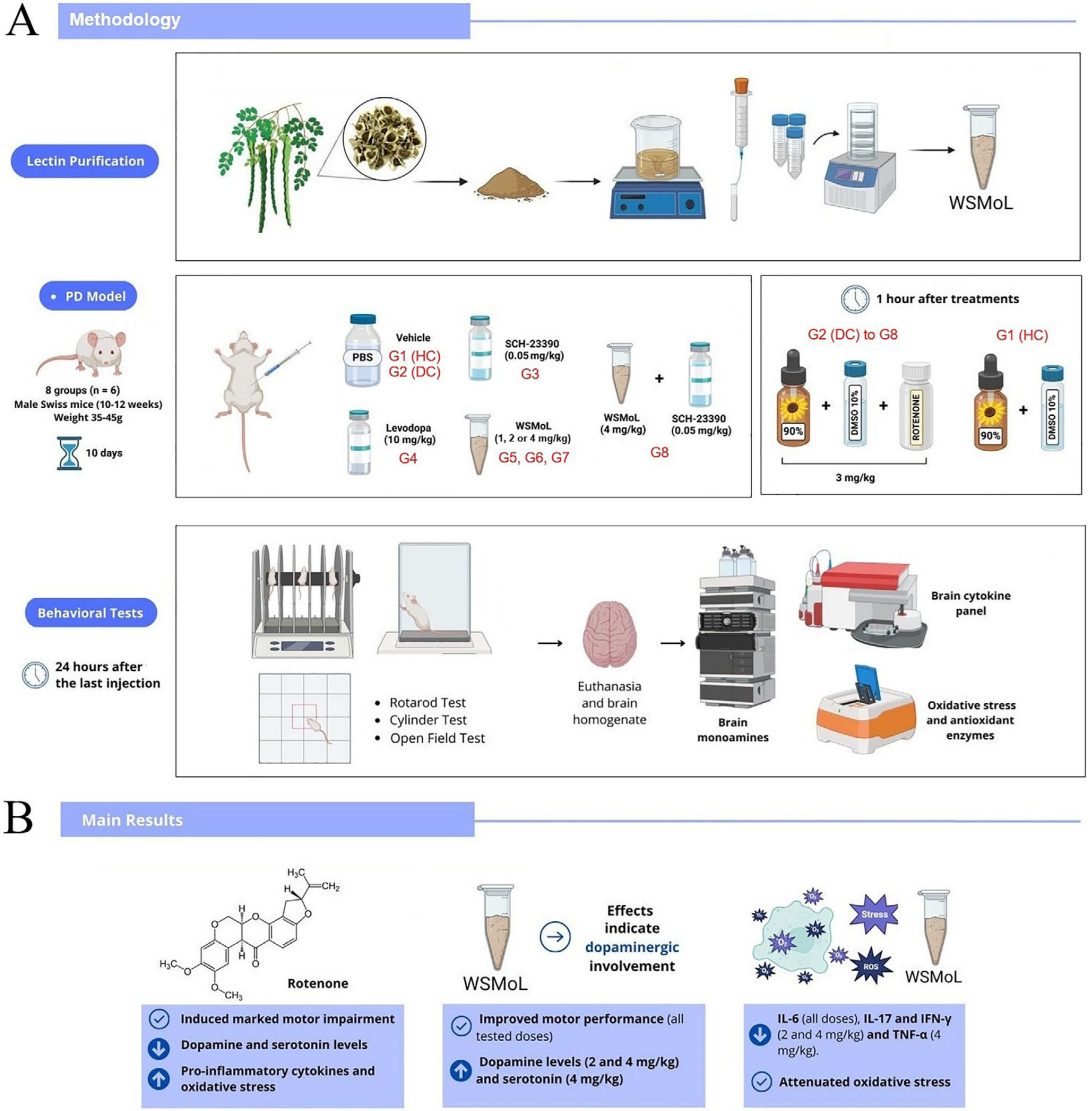
Schematic representation of the experimental design (A) and the main findings (B) from the investigation of the preventive effects of water‐soluble *Moringa oleifera* lectin (WSMoL) on rotenone‐induced Parkinson's disease symptoms in Swiss mice. HC: healthy control; DC: disease control.

Considering the consistent evidence supporting WSMoL as a dopaminergic modulator and its ability to ameliorate dopamine‐deficit‐related conditions, this study aimed to investigate the neuroprotective and anti‐inflammatory effects of this lectin in a rotenone‐induced mouse model of PD. Figure 1A summarizes the experimental design developed to evaluate the hypothesis that WSMoL can prevent rotenone‐induced damage associated with PD‐like symptoms.

## 2 Materials and Methods

### 2.1 Purification of WSMoL

*M. oleifera* seeds were collected from the Experimental Agroforestry System (SAFE) of the Universidade Federal de Pernambuco (UFPE), Recife, Pernambuco, Brazil, under authorization no. 72024 (Instituto Chico Mendes de Conservação da Biodiversidade, ICMBio). Taxonomic identification was performed by Dr. Rita Pereira and a voucher specimen (no. 63,184) is deposited in the herbarium Dárdano de Andrade Lima of the *Instituto Agronômico de Pernambuco* (https://specieslink.net/rec/148/63184). The research was registered (ACC50AF) in the *Sistema Nacional de Gestão do Patrimônio Genético e do Conhecimento Tradicional Associado* (SisGen). WSMoL was isolated as described by Coelho et al. [22], involving protein extraction in distilled water, followed by protein precipitation with ammonium sulfate and purification using chitin affinity chromatography. The lectin was concentrated by freeze‐drying (−50°C, 48 h), and protein content was quantified using the method of Lowry et al. [23], with bovine serum albumin (BSA) as the standard. Hemagglutinating activity was assessed as described by Patriota et al. [24].

### 2.2 Animals

Adult male Swiss mice, aged 10–12 weeks and weighing 35–45 g, obtained from the *Instituto Keizo Asami* (iLIKA) of UFPE, were used in this study. The animals were housed in a climatized vivarium (22°C–26°C) under a 12 h:12 h light–dark cycle. Food and fresh water were provided ad libitum. All experimental procedures were approved by the Ethics Committee on Animal Use of UFPE under protocol no. 0033/2023. The mice were randomly assigned to eight experimental groups using a simple randomization method, with six animals per group (*n* = 6) housed per cage. Throughout the experiment, none of the animals exhibited signs of toxicity that met humane endpoint criteria, therefore, no animals were excluded.

### 2.3 Experimental Parkinsonism Induced by Rotenone

The eight groups (*n* = 6 per group) and respective treatments were as follows: (G1) healthy control (HC, vehicle), (G2) disease control (DC, vehicle), (G3) D1 dopamine receptor antagonist control (SCH‐23390, 0.05 mg/kg, i.p.), (G4) levodopa (L‐DOPA, 10 mg/kg, i.p.), (G5–G7) WSMoL (1, 2, or 4 mg/kg, i.p.), and (G8) SCH‐23390 (0.05 mg/kg, i.p.) co‐administered with WSMoL (4 mg/kg, i.p.) (Figure 1A). Rotenone (Sigma‐Aldrich, St. Louis, MO, USA) was dissolved in a 90% (v/v) sunflower oil and 10% (v/v) dimethyl sulfoxide (DMSO) solution [25] and administered intraperitoneally (i.p.) at 3 mg/kg for 10 days to all groups, except HC, 1 h after the respective treatments. Phosphate‐buffered saline (PBS) was used as the vehicle for WSMoL and other treatments, while the rotenone vehicle consisted of 90% sunflower oil and 10% DMSO. The HC group received vehicle in both administration moments, replacing both rotenone and treatments. To control for potential confounding variables, experimental treatments were always performed in the order described. Group allocation was concealed from investigators, except for the researcher responsible for administering the treatments at each stage. The sample size was defined based on previous studies from our research group investigating the neurobehavioral effects of lectins. Since this is the first study evaluating lectins in a rotenone‐induced PD model, no model‐specific a priori sample size calculation was performed. The selected sample size also complied with the 3Rs principles, minimizing animal use while maintaining an adequate balance between experimental reliability and reduction of animal use.

### 2.4 Monitoring of Body Weight and Water and Food Consumption

Starting from day one of the model, each cage was provided daily with 200 mL of water and 100 g of food, with quantities standardized to allow monitoring of consumption. Water intake was measured using a graduated cylinder, and food consumption was recorded using a semi‐analytical balance. Mice were weighed daily to track changes in body weight. Weight variation was determined by calculating the difference between the mean final and initial body weights of the animals. Relative food and water intake were expressed as a percentage normalized to body weight, using the formula: (intake [g or mL] / body weight [g]) × 100.

### 2.5 Neurobehavioral Tests

Twenty‐after the last rotenone injection (day 11), the behavioral tests described in the following sections were performed to assess motor function.

#### 2.5.1 Rotarod Test

Motor coordination and balance were evaluated using the rotarod test. Mice were placed on a rotating rod set at a constant speed of 18 rpm for a maximum duration of 180 s. The latency to the first fall (i.e., the time elapsed from placement on the rod until the first fall) and the total time spent on the apparatus were recorded [26].

#### 2.5.2 Open Field Test

Locomotor activity was assessed using the open field test. Mice were placed in the center of an open‐field arena (72 × 72 cm) with a grid‐divided floor and allowed to explore freely for 5 min. The total number of quadrant crossings (ambulation frequency) and the number of stops were recorded. The activity index was calculated by dividing the total number of squares crossed by the number of stops, providing an estimate of locomotor activity and movement pattern [27].

#### 2.5.3 Cylinder Test

Forelimb use and motor asymmetry were assessed using the cylinder test. Each mouse was individually placed in the center of a transparent acrylic cylinder (12 cm in diameter, 34 cm in height). The number of forepaw contacts with the cylinder walls over a 5 min period was recorded, without distinguishing between left and right forelimb use [28].

### 2.6 Biochemical Analysis

Mice were euthanized by anesthetic overdose via intraperitoneal administration of ketamine (200 mg/kg) and xylazine (20 mg/kg). Subsequently, craniotomy was performed for brain removal. Each brain was immediately sectioned, rinsed with PBS, and homogenized in 2 mL of PBS supplemented with a protease inhibitor cocktail (Sigma‐Aldrich, St. Louis, MO, USA) using a vortex mixer. The homogenates were centrifuged at 1600 × *g* for 5 min, and the resulting supernatants were collected. Following centrifugation, 1 mL aliquots of each supernatant were processed for dopamine, noradrenaline, and serotonin determination by HPLC‐UV as described in Section 2.6.1. The remaining supernatants were used for protein determination according to Bradford [29], aliquoted, and stored at −80 °C for subsequent biochemical and cytokine analyses.

#### 2.6.1 Quantitative Analysis of Brain Monoamines

Brain homogenate extracts were analyzed for dopamine, noradrenaline, and serotonin concentrations using high‐performance liquid chromatography with ultraviolet detection (HPLC‐UV). After collection of the supernatants described above, 1‐mL aliquots were subjected to solid‐phase extraction using preconditioned C18 extraction columns (500 mg/6 mL; United Chemical Technologies, USA), eluted with 2 mL of 0.11% phosphoric acid/acetonitrile (95:5, v/v), and filtered through a 0.22 µm membrane. The filtered samples were then analyzed using a HPLC system (Shimadzu Corp., Kyoto, Japan) equipped with a Hewlett Packard (HP) 1050 Diode Array Detector. Chromatographic separation was performed in reverse‐phase mode using a sequential arrangement of Zorbax C8 (4.6 × 150 mm, 3.5 µm; Agilent Technologies, Santa Clara, CA, USA) and Shim‐pack C18 (4.6 × 250 mm, 5 µm; Shimadzu Corp.) analytical columns. Detection was carried out at 280 nm, with an injection volume of 50 µL and a flow rate of 1 mL/min of 0.11% phosphoric acid/acetonitrile (95:5, v/v). Standard curves (3–20 mg/L) were used for simultaneous quantification of all analytes. The obtained values represent monoamine concentrations in the analyzed brain homogenate extracts and are expressed as mg/L according to the calibration curves generated with analytical standards. Chromatographic‐grade reagents and standards (Sigma‐Aldrich) were used throughout the analysis.

The analytical performance of the HPLC‐UV method for the determination of dopamine, noradrenaline, and serotonin concentrations was evaluated based on linearity, limits of detection (LOD), limits of quantification (LOQ), and intra‐day precision (repeatability). Dopamine showed a linear response with the regression equation *y* = 48,406.45x−4,364.28 and R^2^ = 0.9950. The estimated LOD and LOQ were 0.90 and 2.73 mg/L, respectively. For serotonin, the calibration curve showed linearity with the equation *y* = 48,650.49x + 1961.08 and R^2^ = 0.9955, while the estimated LOD and LOQ were 0.93 and 2.81 mg/L, respectively. Noradrenaline also exhibited adequate linearity with the regression equation *y* = 31,997.65x−3088.06 and R^2^ = 0.9954, with LOD and LOQ values of 0.89 and 2.69 mg/L, respectively. The intra‐day precision showed low variability among replicate injections of the same extracts, with coefficients of variation of 3.55%, 2.23%, and 3.41% for dopamine, serotonin, and noradrenaline, respectively. Figure S1 presents a representative chromatogram showing the separation of dopamine, noradrenaline, and serotonin under HPLC‐UV conditions.

#### 2.6.2 Brain Cytokine Profiling

Cytokine quantification was performed using the BD Cytometric Bead Array (CBA) Mouse Th1/Th2/Th17 Kit (BD Biosciences, Franklin Lakes, NJ, USA) for the detection of interferon‐gamma (IFN‐γ), interleukins (IL‐2, IL‐4, IL‐6, IL‐10, and IL‐17A), and tumor necrosis factor‐alpha (TNF‐α). The assays were conducted according to the manufacturer's instructions, and data were acquired using an Accuri C6 flow cytometer (BD Biosciences). Individual cytokine standard curves (0–5000 pg/mL) were generated for each assay, with a detection range of 2–5000 pg/mL. Results were analyzed using the BD Accuri C6 software.

#### 2.6.3 Assessment of Oxidative Stress and Antioxidant Enzymes in Brain Homogenates

Catalase activity was determined by monitoring the decrease in absorbance associated with the decomposition of H_2_O_2_ into water, as described by Aebi [30]. Superoxide dismutase (SOD) activity was estimated based on the inhibition of epinephrine oxidation and subsequent adrenochrome formation, following the method of Misra and Fridovich [31]. Lipid peroxidation levels were evaluated by quantifying thiobarbituric acid reactive substances (TBARS), according to Ohkawa et al. [32]. Reduced glutathione (GSH) levels were assessed by measuring non‐protein sulfhydryl groups in the tissue, as described by Sedlak and Lindsay [33]. Basal superoxide production and NADPH oxidase activity were determined using lucigenin‐enhanced chemiluminescence, following previously described procedures [34].

### 2.7 Statistical Analysis

Data are presented as the mean ± standard error of the mean (SEM). Statistical comparisons between experimental and control groups were performed after assessing data normality using the Shapiro–Wilk test, followed by one‐way analysis of variance (ANOVA) and Tukey's post hoc test. A *p*‐value < 0.05, adjusted using the Benjamini–Hochberg false discovery rate (FDR) method, was considered statistically significant. All analyses were performed using GraphPad Prism version 11.0.2.92 (GraphPad Software, La Jolla, CA, USA).

## 3 Results

### 3.1 WSMoL Ameliorated Motor Deficits Induced by Rotenone via Dopaminergic Signaling

**FIGURE 2:**
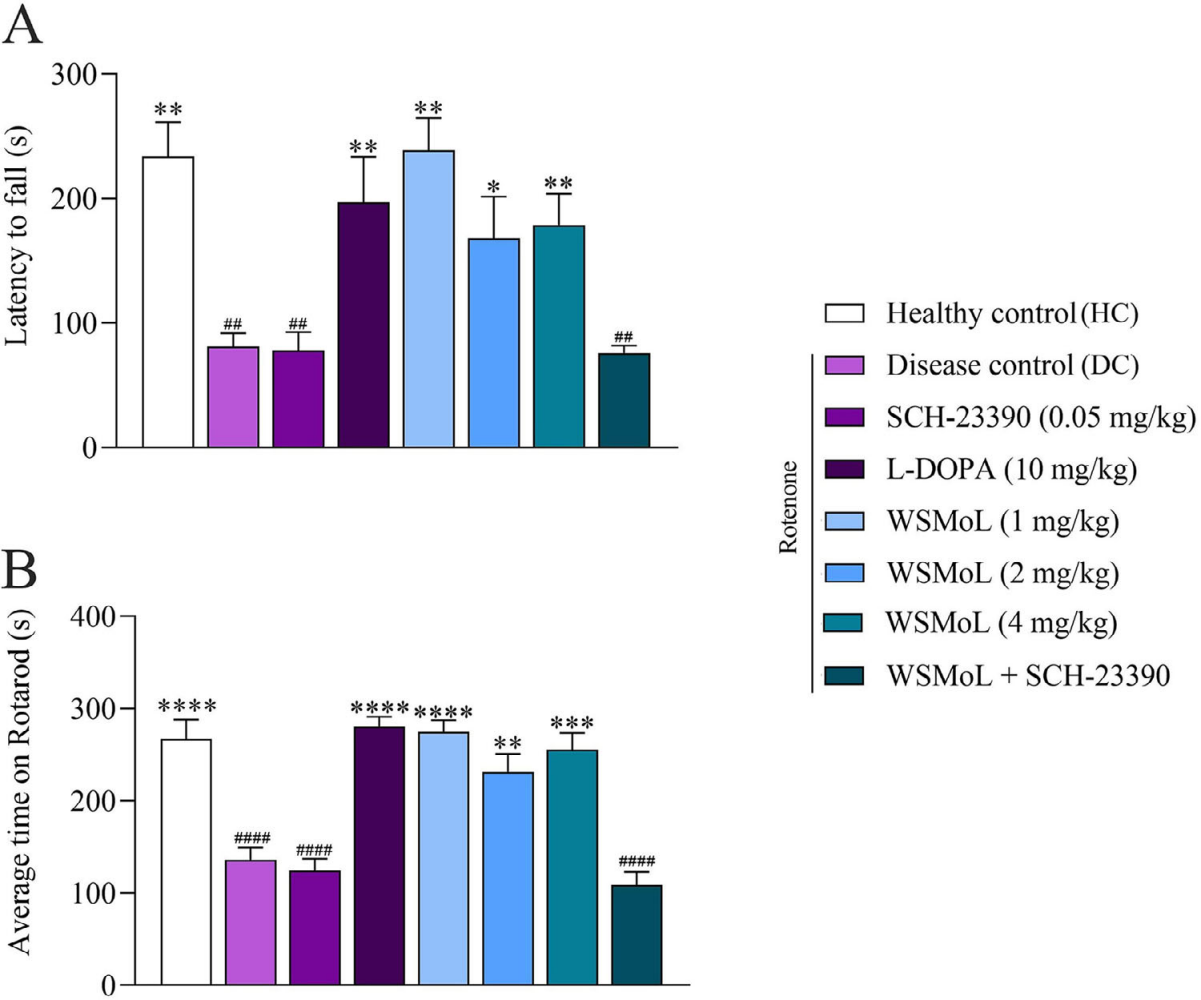
Evaluation of motor behavior using the rotarod test in healthy control (HC) mice or mice subjected to daily rotenone administration (3 mg/kg, i.p.) for 10 days, 1 h after intraperitoneal treatment with vehicle (DC, disease control), SCH‐23390 (0.05 mg/kg), levodopa (L‐DOPA, 10 mg/kg), WSMoL (1, 2, or 4 mg/kg), or SCH‐23390 (0.05 mg/kg) combined with WSMoL (4 mg/kg), being *n* = 6 per group. The latency to fall (A) and the average time spent on the apparatus (B) were recorded. Data are presented as mean ± SEM. Statistical significance was determined by one‐way ANOVA followed by Tukey's post hoc test. Differences were considered significant at *p* < 0.05. Asterisks (*p < 0.05, **p < 0.01, ***p < 0.001, and ****p < 0.0001) indicate comparisons with the DC group, whereas hash symbols (#*p* < 0.05, ##*p* < 0.01, ###*p* < 0.001, and ####*p* < 0.0001) indicate comparisons with the HC group.

Concerning the rotarod test, Figure 2 shows that DC mice exhibited a significant reduction in both the latency to the first fall (Figure 2A; F_7,40_ = 7.732, *p* < 0.0001) and the total time spent on the apparatus (Figure 2B; F_7,40_ = 22.28, *p* < 0.0001) compared to the HC group, indicating that rotenone administration induced motor coordination deficits resembling parkinsonian symptoms. Treatment with L‐DOPA or WSMoL (all doses) significantly protected the mice from developing these deficits, maintaining both the latency to the first fall and the total time on the apparatus at levels comparable to those of the HC group. Notably, co‐administration of the dopamine D1 receptor antagonist SCH‐23390 prevented the protective effects of WSMoL, as both latency and total time remained similar to those observed in the DC group. Additionally, the SCH‐23390 control group did not differ from the DC group.

**FIGURE 3:**
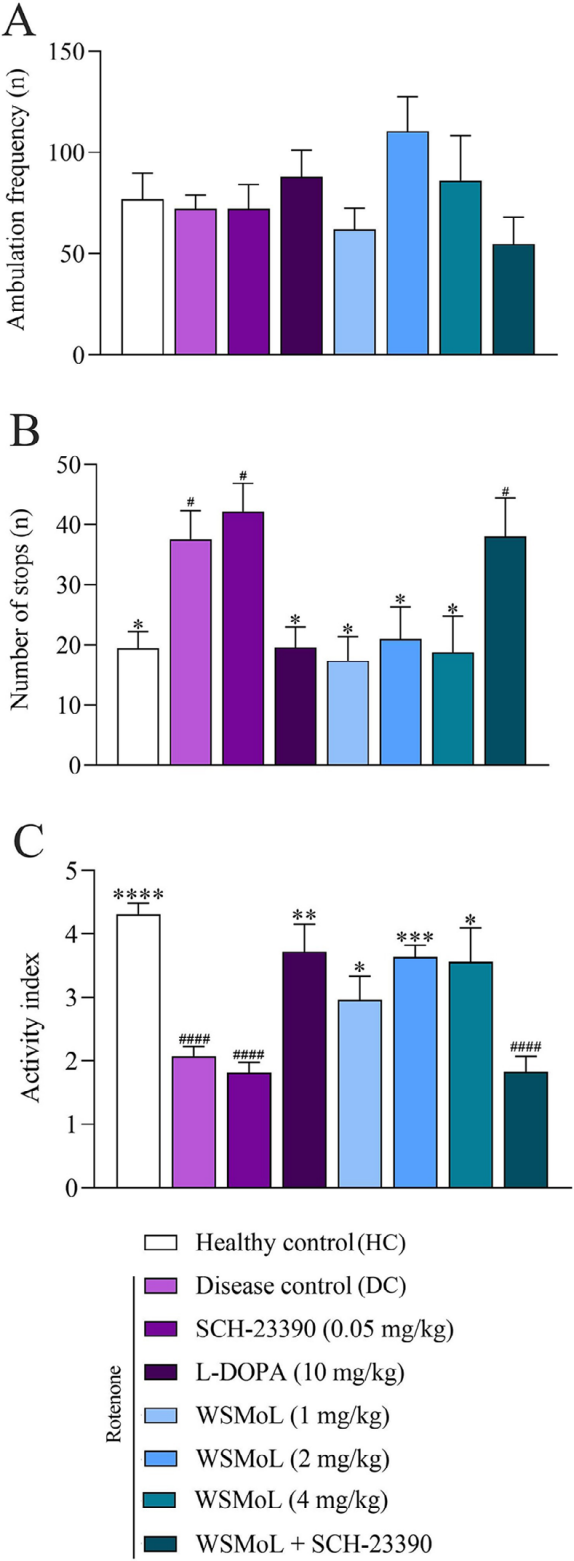
Evaluation of motor behavior using the open field test in healthy control (HC) mice or mice subjected to daily rotenone administration (3 mg/kg, i.p.) for 10 days, 1 h after intraperitoneal treatment with vehicle (DC, disease control), SCH‐23390 (0.05 mg/kg), levodopa (L‐DOPA, 10 mg/kg), WSMoL (1, 2, or 4 mg/kg), or SCH‐23390 (0.05 mg/kg) combined with WSMoL (4 mg/kg), being *n* = 6 per group. Ambulation frequency (A) and the number of stops (B) were recorded. The activity index (C) was calculated as the ratio between ambulation frequency and the number of stops. Data are presented as mean ± SEM. Statistical significance was determined by one‐way ANOVA followed by Tukey's post hoc test. Differences were considered significant at *p* < 0.05. Asterisks (*p < 0.05, **p < 0.01, ***p < 0.001, and ****p < 0.0001) indicate comparisons with the DC group, whereas hash symbols (#*p* < 0.05, ##*p* < 0.01, ###*p* < 0.001, and ####*p* < 0.0001) indicate comparisons with the HC group.

The results of the open field test are presented in Figure 3. No significant differences in ambulation frequency were observed among the experimental groups (Figure 3A; F_7,40_ = 1.476, *p* = 0.2110). However, DC mice exhibited impaired motor performance, as indicated by an increased number of stops (Figure 3B; F_7,40_ = 4.887, *p* = 0.0008) and a reduced activity index (Figure 3C; F_7,40_ = 9.947, *p* < 0.0001) compared to the HC group. In the groups treated with L‐DOPA or WSMoL (all doses), these two parameters were similar to those observed in the HC group, indicating preserved motor function. The co‐administration of SCH‐23390 together with WSMoL abolished the beneficial effects of the lectin on both the number of stops and the activity index, with values remaining statistically similar to those of the DC group. As expected, the SCH‐23390 control group did not differ from the DC group.

**FIGURE 4:**
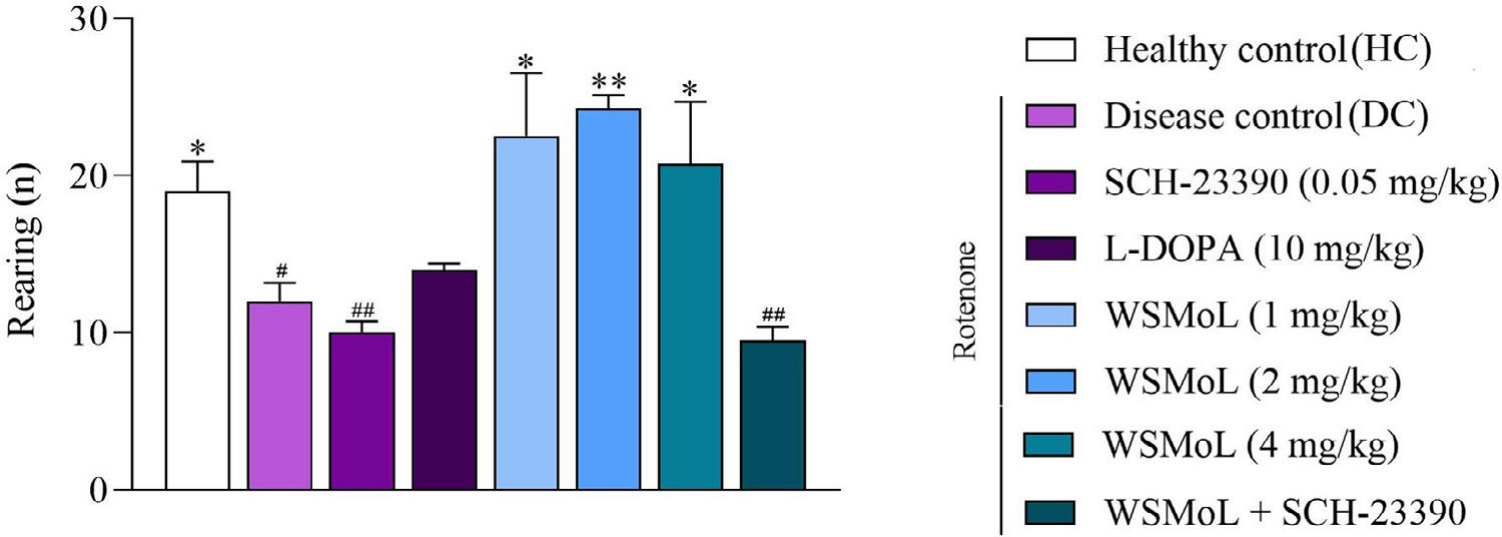
Evaluation of motor behavior using the cylinder test in healthy control (HC) mice or mice subjected to daily rotenone administration (3 mg/kg, i.p.) for 10 days, 1 h after intraperitoneal treatment with vehicle (DC, disease control), SCH‐23390 (0.05 mg/kg), levodopa (L‐DOPA, 10 mg/kg), WSMoL (1, 2, or 4 mg/kg), or SCH‐23390 (0.05 mg/kg) combined with WSMoL (4 mg/kg), being *n* = 6 per group. The number of rearing events was recorded. Data are presented as mean ± SEM. Statistical significance was determined by one‐way ANOVA followed by Tukey's post hoc test. Differences were considered significant at *p* < 0.05. Asterisks (*p < 0.05, **p < 0.01) indicate comparisons with the DC group, whereas hash symbols (#*p* < 0.05, ##*p* < 0.01) indicate comparisons with the HC group.

In the cylinder test (Figure 4; F_7,40_ = 7.015, *p* = 0.0001), DC mice exhibited significantly decreased rearing behavior compared to the HC group, indicating that rotenone reduced the use of the contralateral forelimb and caused motor asymmetry as well as impaired sensorimotor integration. Treatment with WSMoL (all doses) prevented rotenone‐induced impairment of forelimb use, with symmetry levels comparable to those of the HC group. Similar to what was observed in the other tests, co‐administration of SCH‐23390 abolished the beneficial effects of WSMoL, with animals maintaining the motor asymmetry induced by rotenone. The SCH‐23390 control group did not differ from the DC group. In this test, L‐DOPA did not differ statistically from either the DC or HC groups.

### 3.2 WSMoL Prevented the Decrease of Brain Dopamine and Serotonin Levels in Rotenone‐Treated Mice

**FIGURE 5:**
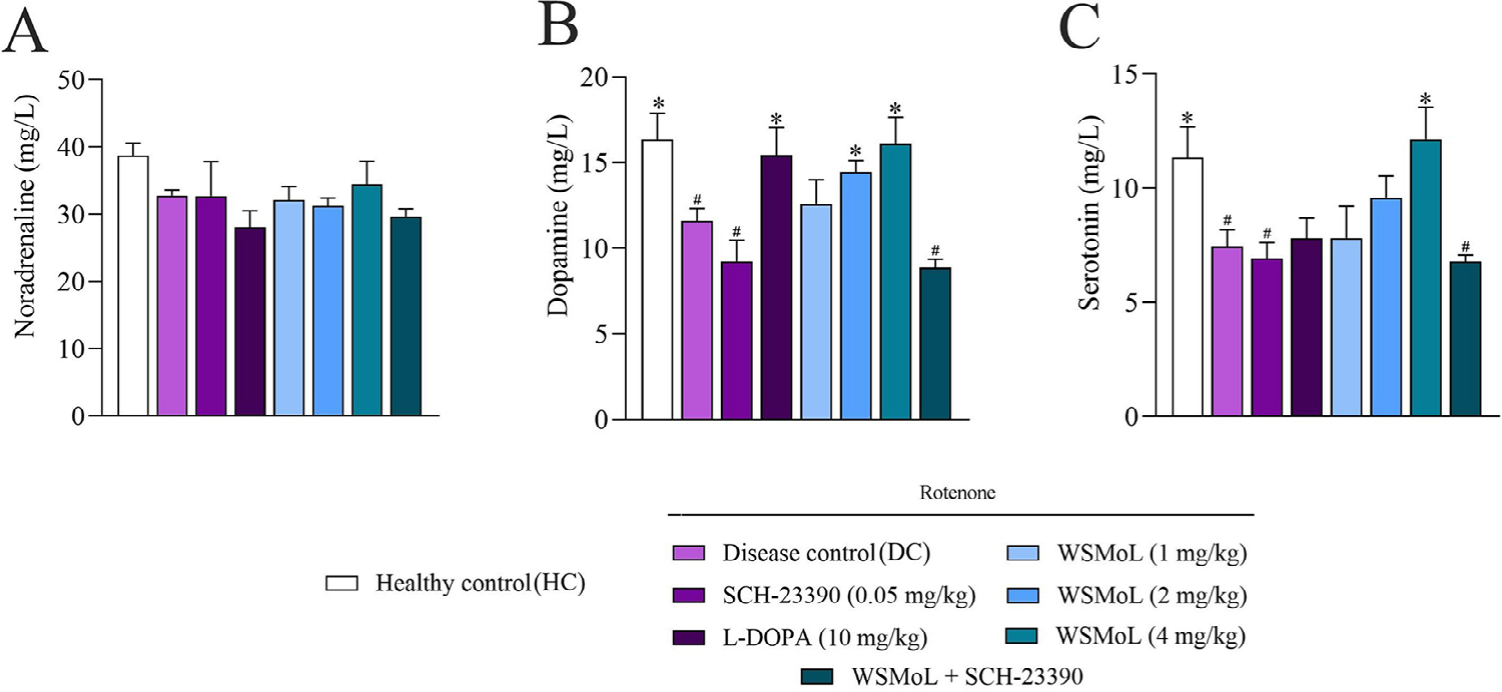
Noradrenaline (A), dopamine (B), and serotonin (C) concentrations in brain homogenate extracts from healthy control (HC) mice or mice subjected to daily rotenone administration (3 mg/kg, i.p.) for 10 days, 1 h after intraperitoneal treatment with vehicle (DC, disease control), SCH‐23390 (0.05 mg/kg), levodopa (L‐DOPA, 10 mg/kg), WSMoL (1, 2, or 4 mg/kg), or SCH‐23390 (0.05 mg/kg) combined with WSMoL (4 mg/kg), being *n* = 6 per group. Data are presented as mean ± SEM. Statistical significance was determined by one‐way ANOVA followed by Tukey's post hoc test. Differences were considered significant at *p* < 0.05. Asterisks (*p < 0.05) indicate comparisons with the DC group, whereas hash symbols (#*p* < 0.05) indicate comparisons with the HC group.

Figure 5 presents neurotransmitter concentrations in the brain homogenate extracts of control and experimental mice. Noradrenaline concentrations did not differ among the groups (Figure 5A; F_7,40_ = 2.816, *p* = 0.0623). Dopamine concentrations (Figure 5B; F_7,40_ = 5.812, *p* = 0.0005) were significantly reduced in DC mice compared to the HC group. This reduction was not observed in mice treated with L‐DOPA or WSMoL (2 and 4 mg/kg), which showed dopamine levels similar to those observed in HC animals. However, this preventive effect was abolished in the group receiving co‐administration of WSMoL and SCH‐23390. Serotonin concentrations (Figure 5C; F_7,40_ = 3.397, *p* = 0.0094) were also decreased in DC mice relative to HC animals. Animals treated with WSMoL at 4 mg/kg exhibited serotonin concentrations comparable to those of the HC group, whereas co‐administration with SCH‐23390 prevented this effect.

### 3.3 WSMoL Prevented Neuroinflammation Caused by Rotenone

**FIGURE 6:**
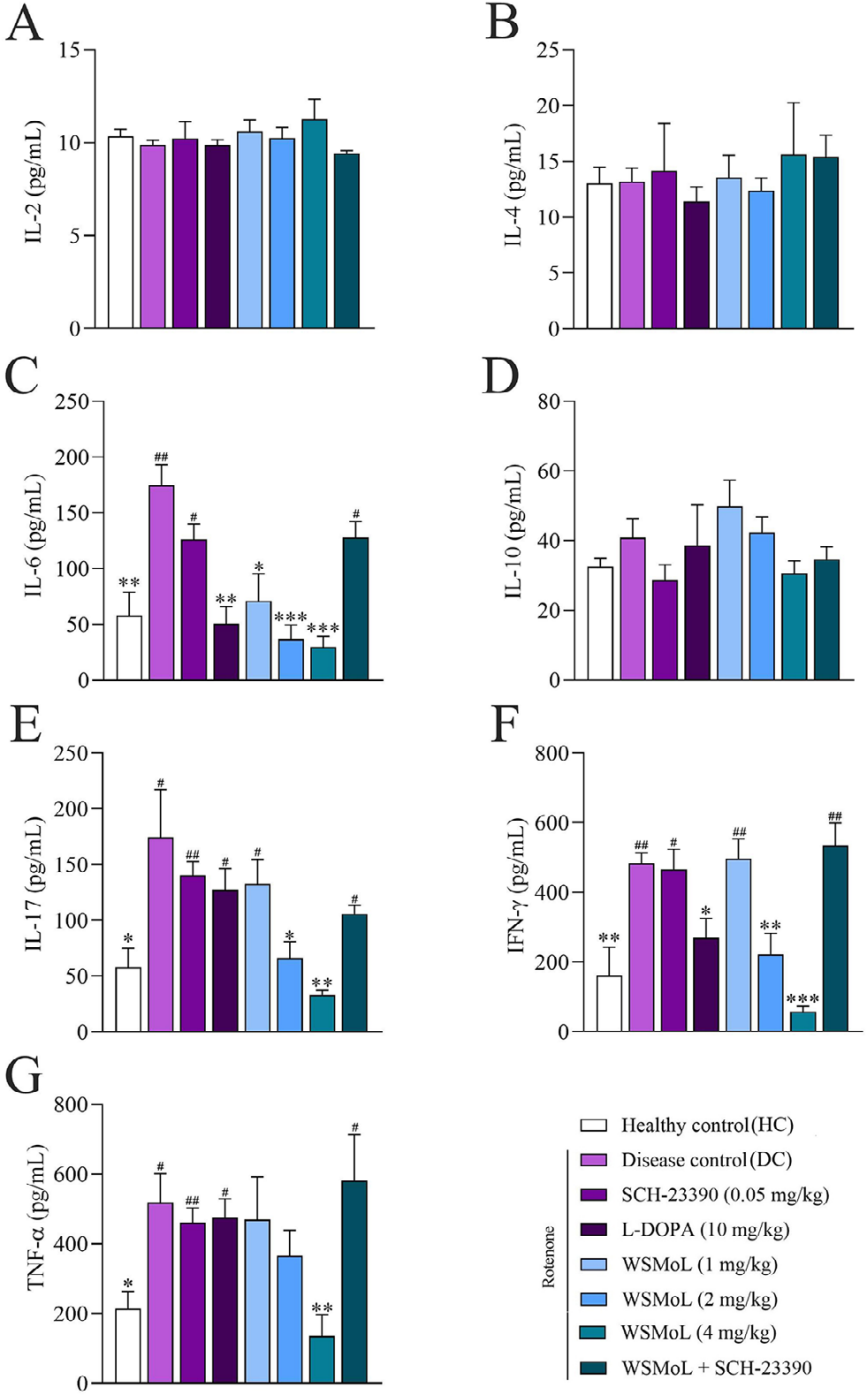
Cytokine levels in brain homogenates from healthy control (HC) mice or mice subjected to daily rotenone administration (3 mg/kg, i.p.) for 10 days, 1 h after intraperitoneal treatment with vehicle (DC, disease control), SCH‐23390 (0.05 mg/kg), levodopa (L‐DOPA, 10 mg/kg), WSMoL (1, 2, or 4 mg/kg), or SCH‐23390 (0.05 mg/kg) combined with WSMoL (4 mg/kg), being *n* = 6 per group. The levels of interleukin (IL)‐2 (A), IL‐4 (B), IL‐6 (C), IL‐10 (D), IL‐17 (E), interferon‐γ (F), and tumor necrosis factor‐α (G) were determined. Data are presented as mean ± SEM. Statistical significance was determined by one‐way ANOVA followed by Tukey's post hoc test. Differences were considered significant at *p* < 0.05. Asterisks (*p < 0.05, **p < 0.01, and ***p < 0.001) indicate comparisons with the DC group, whereas hash symbols (#*p* < 0.05, ##*p* < 0.01, and ###*p* < 0.001) indicate comparisons with the HC group.

Cytokine levels in the brain were measured to elucidate the inflammatory signaling underlying the effects of rotenone administered alone or together with WSMoL. No significant differences were observed in IL‐2 (Figure 6A; F_7,40_ = 0.9200, *p* = 0.5026), IL‐4 (Figure 6B; F_7,40_ = 0.3065, *p* = 0.9440), or IL‐10 (Figure 6D; F_7,40_ = 1.365, *p* = 0.2648) levels among the groups. In contrast, rotenone administration significantly increased the levels in DC mice of IL‐6 (Figure 6C; F_7,40_ = 7.264, *p* = 0.0001), IL‐17A (Figure 6E; F_7,40_ = 5.459, *p* = 0.0008), IFN‐γ (Figure 6F; F_7,40_ = 10.35, *p* < 0.0001), and TNF‐α (Figure 6G; F_7,40_ = 3.392, *p* = 0.0116) compared to the HC group. Treatment with L‐DOPA or WSMoL (all doses) resulted in significantly lower IL‐6 levels than those observed in the DC group, with values comparable to those of the HC group. Regarding IL‐17A, only mice treated with WSMoL at 2 and 4 mg/kg showed levels significantly lower than those of the DC group and similar to those of the HC group, whereas L‐DOPA and WSMoL at 1 mg/kg failed to prevent the rotenone‐induced increase in IL‐17A. IFN‐γ levels were significantly lower in groups treated with L‐DOPA or WSMoL at 2 and 4 mg/kg compared to DC mice, reaching levels comparable to those of the HC group, while WSMoL at 1 mg/kg did not produce this effect. A significantly lower TNF‐α level was observed only at the highest dose of WSMoL, whereas the L‐DOPA‐treated group did not differ statistically from the DC group. Notably, co‐administration with the dopamine D1 receptor antagonist SCH‐23390 prevented the anti‐inflammatory effects of WSMoL.

### 3.4 WSMoL Mitigated Oxidative Stress Caused by Rotenone Maintaining the Antioxidant Defenses

**FIGURE 7:**
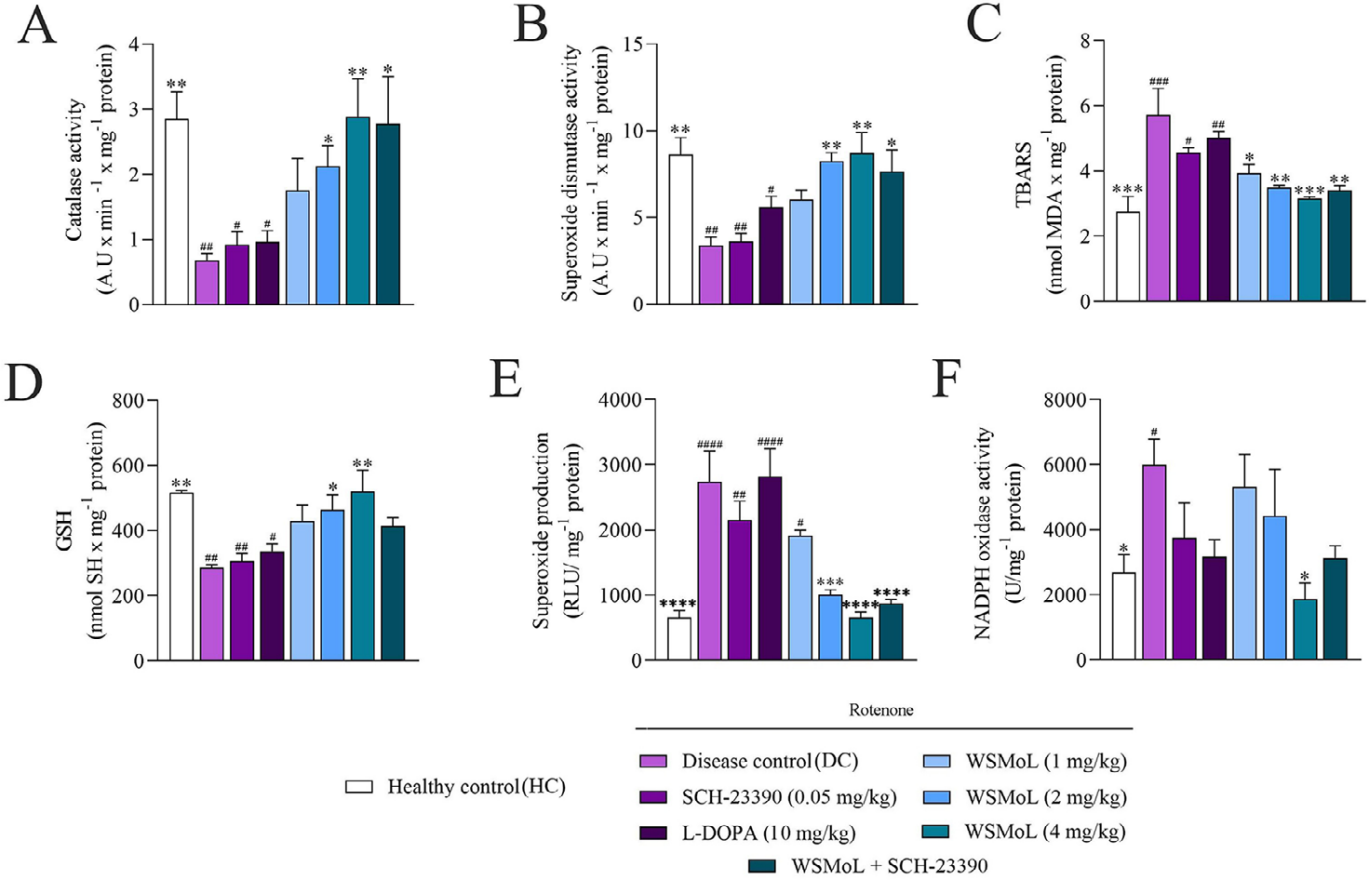
Levels of oxidative stress indicators in brain homogenates from healthy control (HC) mice or mice subjected to daily rotenone administration (3 mg/kg, i.p.) for 10 days, 1 h after intraperitoneal treatment with vehicle (DC, disease control), SCH‐23390 (0.05 mg/kg), levodopa (L‐DOPA, 10 mg/kg), WSMoL (1, 2, or 4 mg/kg), or SCH‐23390 (0.05 mg/kg) combined with WSMoL (4 mg/kg), being *n* = 6 per group. Catalase activity (A), superoxide dismutase activity (B), thiobarbituric acid reactive substances (TBARS) (C), reduced glutathione (GSH) levels (D), superoxide production (E), and NADPH oxidase activity (F) were assessed. Data are presented as mean ± SEM. Statistical significance was determined by one‐way ANOVA followed by Tukey's post hoc test. Differences were considered significant at *p* < 0.05. Asterisks (*p < 0.05, **p < 0.01, ***p < 0.001, ****p < 0.0001) indicate comparisons with the DC group, whereas hash symbols (#*p* < 0.05, ##*p* < 0.01, ###*p* < 0.001, and ####*p* < 0.0001) indicate comparisons with the HC group.

Brain catalase (Figure 7A; F_7,40_ = 4.800, *p* = 0.0007) and superoxide dismutase activities (Figure 7B; F_7,40_ = 7.032, *p* < 0.0001) were significantly decreased in DC mice compared to the HC group. Treatment with WSMoL (2 and 4 mg/kg), as well as co‐administration of WSMoL + SCH‐23390, maintained the activities of these enzymes at levels comparable to those observed in HC animals.

Lipid peroxidation (Figure 7C; F_7,40_ = 7.789, *p* < 0.0001) was markedly elevated in the DC group relative to HC mice. In contrast, treatment with WSMoL (1, 2, and 4 mg/kg) and WSMoL + SCH‐23390 prevented the rotenone‐induced increase in lipid peroxidation, resulting in values similar to those of the HC group. Regarding reduced glutathione (GSH) levels (Figure 7D; F_7,40_ = 5.836, *p* = 0.0002), DC mice exhibited significant depletion compared to HC animals. Administration of WSMoL at 2 and 4 mg/kg maintained GSH levels comparable to those of the HC group and significantly higher than those observed in DC mice.

Superoxide production (Figure 7E; F_7,40_ = 13.48, *p* < 0.0001) was significantly increased in DC animals relative to the HC group. Treatment with WSMoL (2 and 4 mg/kg) and WSMoL + SCH‐23390 maintained superoxide levels comparable to those of HC mice and significantly lower than those of DC mice. Similarly, NADPH oxidase activity (Figure 7F; F_7,40_ = 2.699, *p* = 0.0230) was elevated in DC mice compared to HC animals. Among the treatments, only WSMoL at 4 mg/kg maintained NADPH oxidase activity at levels comparable to those of the HC group and lower than those observed in DC mice.

Overall, L‐DOPA exhibited a pro‐oxidant profile, with outcomes resembling those of DC mice and differing significantly from the HC group across the evaluated parameters.

### 3.5 Body Weight, Food, and Water Intake

**FIGURE 8:**
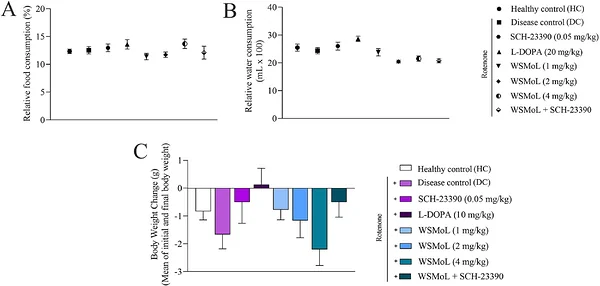
Relative consumption of food (A) and water (B) and body weight change (C) of healthy control (HC) mice or mice subjected to daily rotenone administration (3 mg/kg, i.p.) for 10 days, 1 h after intraperitoneal treatment with vehicle (DC, disease control), SCH‐23390 (0.05 mg/kg), levodopa (L‐DOPA, 10 mg/kg), WSMoL (1, 2, or 4 mg/kg), or SCH‐23390 (0.05 mg/kg) combined with WSMoL (4 mg/kg), being *n* = 6 per group. Data are presented as mean ± SEM. No statistically significant difference was detected using one‐way ANOVA followed by Tukey's post hoc test in comparisons with the DC group or HC group.

As shown in Figure 8, no significant differences were observed in food intake (Figure 8A; F_7,40_ = 1.234, *p* = 0.2975), water intake (Figure 8B; F_7,40_ = 2.860, *p* = 0.0837), or body weight (Figure 8C; F_7,40_ = 2.086, *p* = 0.0683) among the HC, DC, and treatment groups.

## 4 Discussion

In this study, it was demonstrated that the lectin WSMoL significantly attenuated rotenone‐induced motor impairments in mice, which are characteristic of PD‐like symptoms. Notably, this neuroprotective effect was consistently observed across all tested doses and was comparable to that achieved with L‐DOPA, the gold‐standard antiparkinsonian therapy.

The co‐administration of the dopamine D1 receptor antagonist SCH‐23390 attenuated several beneficial effects of WSMoL, suggesting that dopaminergic signaling contributes to its effects. This finding is particularly relevant given that degeneration of dopaminergic neurons in the substantia nigra pars compacta is a hallmark of PD, and the maintenance of dopaminergic signaling is essential for motor function and disease‐related alterations [35, 36]. D1 receptors, predominantly expressed in the striatal direct pathway, play a central role in facilitating motor activity [37] and are key mediators of the therapeutic effects of L‐DOPA [38]. Alterations in D1 receptor signaling have been implicated in both motor and non‐motor manifestations of PD, underscoring their relevance as a pharmacological target [37]. However, considering that SCH‐23390 primarily blocks D1 receptor‐mediated signaling rather than dopamine synthesis, the reduction in dopamine levels observed after co‐administration should be interpreted as a secondary consequence of altered dopaminergic regulation rather than a direct inhibition of dopamine production. Overall, the findings suggest that D1 receptor signaling contributes to the effects of WSMoL, supporting further investigation of this lectin in experimental models of parkinsonism.

Interestingly, in acute screening models for anxiolytic and antidepressant agents, WSMoL has been shown to exert its effects through monoaminergic signaling pathways. The dopamine D1 receptor antagonist SCH‐23390 reversed the anxiolytic‐ and antidepressant‐like effects of WSMoL, supporting the involvement of D1 receptor‐mediated mechanisms [19, 20].

At the molecular level, the analysis of neurotransmitter concentrations (dopamine, serotonin, and noradrenaline) in brain homogenates further elucidated the mechanisms underlying the effects of WSMoL. Treatment with WSMoL resulted in significantly higher dopamine and serotonin concentrations than those observed in the DC group, consistent with its ability to preserve monoaminergic homeostasis under neurotoxic conditions. Similar monoaminergic modulation has been reported by Patriota et al. [21] in mice subjected to the unpredictable chronic mild stress (UCMS) model, in which WSMoL also produced anxiolytic‐ and antidepressant‐like effects, reinforcing the hypothesis that its actions converge on dopamine‐ and serotonin‐dependent pathways across distinct neurobehavioral paradigms. In the present PD model, the maintenance of dopaminergic tone is particularly relevant [39], as it likely explains the preservation of motor performance in WSMoL‐treated animals, which remained comparable to healthy control. This effect may reflect either a prevention of rotenone‐induced dopaminergic depletion or a more rapid re‐establishment of dopamine homeostasis, suggesting a stabilizing action on vulnerable circuits. However, because biochemical analyses were performed using whole‐brain homogenates, regional effects within the nigrostriatal pathway could not be determined. The convergence of findings across both PD‐like and UCMS models further supports the involvement of shared monoaminergic regulatory mechanisms, potentially mediated via D1 receptor‐dependent signaling.

Although α‐synuclein aggregation was not evaluated in the present study, these neurochemical effects raise the possibility that WSMoL may interfere with upstream pathogenic processes relevant to PD, particularly α‐synuclein aggregation, a central feature of dopaminergic neurodegeneration [40]. Given the close relationship between impaired dopamine handling, oxidative stress, and α‐synuclein misfolding, future studies should investigate whether the behavioral and neurochemical protection conferred by WSMoL is accompanied by reduced α‐synuclein aggregation or propagation, thereby clarifying its potential disease‐modifying properties.

Neuroinflammation is increasingly recognized as a central mechanism in PD progression, contributing directly to dopaminergic neuronal loss [36, 41]. In line with this, we observed that rotenone markedly increased brain levels of pro‐inflammatory cytokines, including IL‐6, IL‐17A, IFN‐γ, and TNF‐α, consistent with microglial activation and an exacerbated neuroinflammatory state. WSMoL treatment effectively prevented this inflammatory profile, indicating a strong anti‐inflammatory and neuroprotective action. This anti‐inflammatory effect of WSMoL was abolished by co‐administration of SCH‐23390.

One plausible explanation is that WSMoL preserved dopamine levels in rotenone‐treated animals, thereby maintaining sufficient dopamine levels to engage D1 receptor‐mediated pathways that exert downstream inhibitory control over microglial activation and cytokine production. In this context, dopamine has been reported to regulate neuroimmune responses and limit excessive inflammatory activation in the brain [42]. Thus, when D1 receptors were pharmacologically blocked, even in the presence of WSMoL, this dopaminergic brake on inflammation was lost, and the anti‐inflammatory effect was no longer observed. This interpretation is supported by the parallel behavioral and neurochemical findings, in which SCH‐23390 also prevented WSMoL‐induced improvements in motor performance and dopaminergic restoration. Together, these data suggest that the anti‐inflammatory effects of WSMoL are not independent of its dopaminergic actions, but rather are tightly coupled to the preservation of dopamine signaling. This dual modulation of dopamine homeostasis and neuroinflammation points to a coordinated neuroprotective mechanism in which maintenance of dopaminergic tone is also involved, rather than solely immunomodulation *per se*.

It is well established that chronic microglial activation in PD leads to a persistent pro‐inflammatory environment, which exacerbates neuronal death and creates a vicious cycle of degeneration [43, 44]. By attenuating this microglial response, WSMoL also interrupts this cycle, thereby attenuating biochemical and behavioral alterations associated with dopaminergic dysfunction. The expression of DA receptors by microglia has been well established, including D1‐like receptors [45].

Recombinant galectin‐1 administered to MPTP‐lesioned mice preserved motor function, reduced microglial activation, and decreased the secretion of pro‐inflammatory cytokines, such as IL‐1β and TNF‐α, as well as the expression of inducible nitric oxide synthase and cyclooxygenase‐2 [44]. This agrees with our results that demonstrated that lectins can exert neuroprotective effects in a PD context and are capable of modulating neuroinflammation associated with dopaminergic neurodegeneration. In a similar direction, Patriota et al. [21] showed that WSMoL attenuated the release of pro‐inflammatory cytokines, including IL‐2, TNF‐α, and IL‐6, in both serum and brain tissue in mice subjected to the UCMS model of anxiety and depression.

In addition to neuroinflammation, oxidative stress pathways were also strongly affected by rotenone administration. However, WSMoL prevented the increase of key cellular oxidative stress markers, including superoxide production, lipid peroxidation, and NADPH oxidase activity, while maintaining the availability of the antioxidant defense system (catalase, SOD, and GSH). Interestingly, the administration of SCH‐23390 with WSMoL did not prevent the protective effect of WSMoL at 4 mg/kg regarding these parameters. This dissociation suggests that WSMoL exerts multimodal neuroprotective actions through distinct mechanisms, with D1 receptor‐mediated signaling contributing mainly to its anti‐inflammatory and motor effects, whereas the protective effects against oxidative stress appear to occur through D1‐independent pathways, possibly involving direct modulation of cellular redox homeostasis. In contrast, L‐DOPA exhibited a pro‐oxidant profile, which is explained because, when converted into dopamine by aromatic L‐amino acid decarboxylase, L‐DOPA can produce reactive intermediates that induce damage to lipids, proteins, and DNA, while also consuming endogenous antioxidant defenses, such as SOD, catalase, and GSH [46].

Current PD treatments provide symptomatic relief, but do not halt disease progression and are often associated with long‐term complications, including dyskinesia [11]. The future management of PD will likely rely on the continued use of symptomatic therapies, such as L‐DOPA, alongside the development and investigation of agents with potential disease‐modifying effects [39]. Our findings suggest that WSMoL may offer a broad therapeutic advantage by improving motor function, mitigating inflammation, restoring neurotransmitter balance, and attenuating oxidative stress. Although the precise mechanisms underlying these effects remain to be fully elucidated, the apparent involvement of dopaminergic signaling, particularly through D1 receptor‐mediated pathways, combined with the ability of WSMoL to mitigate neuroinflammation and oxidative stress, supports its potential as a promising candidate for further therapeutic development, either as a standalone strategy or as an adjunct to L‐DOPA, similarly to other dopaminergic agonists currently used in PD therapy.

Previous investigations have examined the acute and subacute toxicity profile of WSMoL in mice. In the acute protocol, administration of a single intraperitoneal dose of 200 mg/kg resulted in 60% mortality, whereas treatment with 100 mg/kg i.p. produced no deaths or significant hematological and histopathological abnormalities [47]. In subacute assays, animals received daily intraperitoneal WSMoL doses of 10, 25, or 50 mg/kg for 14 consecutive days, and the lectin exhibited a generally acceptable safety profile. No mortality, behavioral impairment, or relevant changes in food consumption, body mass, or most biochemical and hematological markers were detected. Nevertheless, reductions in monocyte and lymphocyte counts, together with mild inflammatory alterations in the liver, kidneys, and spleen were reported [48]. Based on these findings, studies investigating the neuropharmacological actions of WSMoL, including the present work, have preferentially employed doses below 5 mg/kg i.p.

Such findings are important for supporting the progression of lectin‐based compounds from experimental studies toward clinical application. Beyond toxicological considerations, Ferreira et al. [49] emphasized that future advances in lectin therapeutics will require strategies to reduce immunogenicity and overcome limitations related to delivery systems and molecular stability. Therefore, additional investigations are warranted to further elucidate the therapeutic potential of WSMoL. In the context of its neuromodulatory activity, future research should also include pharmacokinetic and bioavailability analyses to determine tissue distribution, blood–brain barrier permeability, and the persistence of its biological effects.

Although the rotenone‐induced PD model used in this study successfully reproduced several hallmark alterations associated with the disease, including behavioral deficits, neurochemical imbalance, inflammation, and oxidative stress, complementary experimental approaches may provide a broader understanding of WSMoL activity during neurodegeneration. In particular, chronic and progressive models could help elucidate the long‐term effects of treatment throughout different stages of PD progression. Moreover, histopathological and immunohistochemical evaluations may clarify whether WSMoL contributes to dopaminergic neuron preservation, modulation of microglial responses, or attenuation of α‐synuclein aggregation. Additional analyses focusing on PD‐related brain regions, especially the substantia nigra and striatum, may further improve the understanding of the mechanisms underlying the neuroprotective effects observed in the present study.

## 5 Conclusion

The present study demonstrates, for the first time, the neuroprotective‐like effects of a plant lectin in a PD model (Figure 1B). WSMoL effectively attenuated motor impairments, maintained dopamine and serotonin concentrations in brain homogenate extracts, reduced neuroinflammatory mediators, and mitigated oxidative stress while maintaining endogenous antioxidant defenses. These findings support the concept that WSMoL acts through integrated modulation of monoaminergic signaling, neuroinflammation, and oxidative stress, which are three central processes involved in PD pathophysiology. Importantly, the beneficial effects observed with WSMoL were comparable to, and in some oxidative parameters potentially greater than, those of L‐DOPA treatment. The present data provide evidence supporting the neuroprotective‐like potential of WSMoL in this experimental model and lay the foundation for additional studies aimed at better characterizing its molecular targets, pharmacokinetic profile, and long‐term effects in progressive models of neurodegeneration.

## Author Contributions

**Leydianne Leite de Siqueira Patriota**: conceptualization, data curation, formal analysis, investigation, methodology, project administration, validation, visualization, and writing – original draft. **Emanuel Davi Lima de Matos Leão**: Investigation and methodology. **Maria Nívea Bezerra da Silva**: investigation, methodology, and visualization. **Amanda de Oliveira Marinho**: data curation, investigation, and methodology. **Bárbara Raíssa Ferreira de Lima**: investigation and methodology. **Jainaldo Alves da Costa**: investigation. **Luciclaudio Cassimiro de Amorim**: investigation. **Valéria Bianca de Souza Santos**: data curation, investigation, and methodology. **Patrícia Maria Guedes Paiva**: funding acquisition and resources. **Michelly Cristiny Pereira**: funding acquisition and resources. **Daniella Carla Napoleão**: funding acquisition, methodology, resources, and validation. **Jorge Vinícius Fernandes Lima Cavalcanti**: funding acquisition, methodology, resources, and validation. **Leucio Duarte Vieira**: funding acquisition, methodology, resources, and validation. **Michelle Melgarejo da Rosa**: conceptualization, funding acquisition, methodology, project administration, resources, supervision, validation, visualization, and writing – review & editing. **Thiago Henrique Napoleão**: conceptualization, funding acquisition, methodology, project administration, resources, supervision, validation, visualization, and writing – review & editing.

## Declaration of Generative AI and AI‐Assisted Technologies in the Writing Process

During the preparation of this work the authors used ChatGPT for the purpose of correcting grammatical and spelling errors. After using this tool/service, the authors reviewed and edited the content as needed and take full responsibility for the content of the published article.

## Conflicts of Interest

The authors declare no conflicts of interest.

## Supporting information




**Supporting File 1**: cbdv71646‐sup‐0001‐S1.pdf

## Data Availability

The data that support the findings of this study are available from the corresponding author upon reasonable request.

## References

[1] O.‐B. Tysnes and A. Storstein , “Epidemiology of Parkinson's Disease,” Journal of Neural Transmission 124 (2017): 901–905, 10.1007/s00702-017-1686-y. 28150045

[2] E. Tolosa , A. Garrido , S. W. Scholz , and W. Poewe , “Challenges in the diagnosis of Parkinson's Disease,” Lancet Neurology 20 (2021): 385–397, 10.1016/S1474-4422(21)00030-2. 33894193 PMC8185633

[3] T. N. Pham , R. E. Schelling , and K. H. Loh , “Parkinson's Disease and Metabolic Disorders: Understanding Their Shared Co‐Morbidity Through the Autonomic Nervous System,” Advances in Genetics 113 (2025): 199–247. 40409798 10.1016/bs.adgen.2025.02.001 PMC12707340

[4] S. Rajan and B. Kaas , “Parkinson's Disease: Risk Factor Modification and Prevention,” Seminars in Neurology 42 (2022): 626–638. 36427528 10.1055/s-0042-1758780

[5] S. Kumar , L. Goyal , and S. Singh , “Tremor and Rigidity in Patients With Parkinson's Disease: Emphasis on Epidemiology, Pathophysiology and Contributing Factors,” CNS & Neurological Disorders—Drug Targets 21 (2022): 596–609, 10.2174/1871527320666211006142100. 34620070

[6] C. Raza , R. Anjum , and N. A. Shakeel , “Parkinson's Disease: Mechanisms, Translational Models and Management Strategies,” Life Sciences 226 (2019): 77–90, 10.1016/j.lfs.2019.03.057. 30980848

[7] D. K. Simon , C. M. Tanner , and P. Brundin , “Parkinson Disease Epidemiology, Pathology, Genetics, and Pathophysiology,” Clinics in Geriatric Medicine 36 (2020): 1–12. 31733690 10.1016/j.cger.2019.08.002 PMC6905381

[8] Z. Chen , G. Li , and J. Liu , “Autonomic Dysfunction in Parkinson's Disease: Implications for Pathophysiology, Diagnosis, and Treatment,” Neurobiology of Disease 134 (2020): 104700. 31809788 10.1016/j.nbd.2019.104700

[9] J. Jankovic , “Parkinson's Disease: Clinical Features and Diagnosis,” Journal of Neurology, Neurosurgery & Psychiatry 79 (2008): 368–376, 10.1136/jnnp.2007.131045. 18344392

[10] L. Christopher , Y. Koshimori , A. E. Lang , M. Criaud , and A. P. Strafella , “Uncovering the Role of the Insula in Non‐Motor Symptoms of Parkinson's Disease,” Brain 137 (2014): 2143–2154, 10.1093/brain/awu084. 24736308 PMC4107733

[11] M. A. Cenci , “Presynaptic Mechanisms of L‐DOPA‐Induced Dyskinesia: The Findings, the Debate, and the Therapeutic Implications,” Frontiers in Neurology 5 (2014): 242, 10.3389/fneur.2014.00242. 25566170 PMC4266027

[12] S. Patel , X. Garcia , M. E. Mohammad , et al., “Dopamine Agonist Withdrawal Syndrome (DAWS) in a Tertiary Parkinson Disease Treatment Center,” Journal of the Neurological Sciences 379 (2017): 308–311, 10.1016/j.jns.2017.06.022. 28716269

[13] H. Wilson , G. Pagano , E. R. de Natale , et al., “Mitochondrial Complex 1, Sigma 1, and Synaptic Vesicle 2A in Early Drug‐Naive Parkinson's Disease,” Movement Disorders 35 (2020): 1416–1427, 10.1002/mds.28064. 32347983

[14] M. Lucchesi , L. Biso , M. Bonaso , et al., “Mitochondrial Dysfunction in Genetic and Non‐Genetic Parkinson's Disease,” International Journal of Molecular Sciences 26 (2025): 4451, 10.3390/ijms26094451. 40362688 PMC12072996

[15] M. T. Ibarra‐Gutiérrez , N. Serrano‐García , and M. Orozco‐Ibarra , “Rotenone‐Induced Model of Parkinson's Disease: Beyond Mitochondrial Complex I Inhibition,” Molecular Neurobiology 60 (2023): 1929–1948, 10.1007/s12035-022-03193-8. 36593435

[16] L. Nahar , R. Charoensup , and K. Kalieva , “Natural Products in Neurodegenerative Diseases: Recent Advances and Future Outlook,” Frontiers in Pharmacology 16 (2025): 1529194, 10.3389/fphar.2025.1529194. 40176910 PMC11961910

[17] I. O. Ishola , I. O. Awogbindin , T. G. Olubodun‐Obadun , O. A. Oluwafemi , J. E. Onuelu , and O. O. Adeyemi , “Morin Ameliorates Rotenone‐Induced Parkinson Disease in Mice Through Antioxidation and Anti‐Neuroinflammation: Gut‐Brain Axis Involvement,” Brain Research 1789 (2022): 147958, 10.1016/j.brainres.2022.147958. 35654119

[18] J. R. C. Araújo , C. B. Coelho , A. R. Campos , R. A. Moreira , and A. C. O. Monteiro‐Moreira , “Animal Galectins and Plant Lectins as Tools for Studies in Neurosciences,” Current Neuropharmacology 18 (2020): 202–215. 31622208 10.2174/1570159X17666191016092221 PMC7327950

[19] L. L. S. Patriota , B. R. F. de Lima , A. D. O. Marinho , et al., “The Anxiolytic‐Like Activity of Water‐Soluble *Moringa oleifera* Lam. lectin is Mediated via Serotoninergic, Noradrenergic, and Dopaminergic Neurotransmission,” Brain Disorders 9 (2023): 100066, 10.1016/j.dscb.2023.100066.

[20] L. L. de Siqueira Patriota , B. R. F. de Lima , A. de Oliveira Marinho , et al., “Water‐Soluble *Moringa oleifera* Seed Lectin Exhibits Monoaminergic Pathway‐Linked Anti‐Depressive‐Like Effects in Mice,” Protein & Peptide Letters 30 (2023): 1048–1057, 10.2174/0109298665270366231031052629. 38018205

[21] L. L. de Siqueira Patriota , B. R. F. de Lima , A. de Oliveira Marinho , et al., “ *Moringa oleifera* Lam. Seed Lectin (WSMoL) Reduces Chronic Stress‐Induced Anxiety and Depression in Mice by Lessening Inflammation and Balancing Brain Chemicals,” Behavioural Brain Research 477 (2025): 115318, 10.1016/j.bbr.2024.115318. 39481762

[22] J. S. Coelho , N. D. L. Santos , T. H. Napoleão , et al., “Effect of *Moringa oleifera* Lectin on Development and Mortality of *Aedes aegypti* Larvae,” Chemosphere 77 (2009): 934–938, 10.1016/j.chemosphere.2009.08.022. 19747711

[23] O. H. Lowry , N. J. Rosebrough , A. L. Farr , and R. J. Randall , “Protein Measurement With the Folin Phenol Reagent,” Journal of Biological Chemistry 193 (1951): 265–275, 10.1016/S0021-9258(19)52451-6. 14907713

[24] L. L. de Siqueira Patriota , T. F. Procópio , J. de Santana Brito , et al., “ *Microgramma vacciniifolia* (Polypodiaceae) Fronds Contain a Multifunctional Lectin With Immunomodulatory Properties on Human Cells,” International Journal of Biological Macromolecules 103 (2017): 36–46, 10.1016/j.ijbiomac.2017.05.037. 28501598

[25] G. Swamy , R. Holla , and S. R. Rao , “Establishing the Rotenone‐Induced Parkinson's Disease Animal Model in Wistar Albino Rats,” Journal of Health and Allied Sciences NU 11 (2021): 158–163.

[26] S. A. Zaitone , E. Ahmed , N. M. Elsherbiny , et al., “Caffeic Acid Improves Locomotor Activity and Lessens Inflammatory Burden in a Mouse Model of Rotenone‐Induced Nigral Neurodegeneration: Relevance to Parkinson's Disease Therapy,” Pharmacological Reports 71 (2019): 32–41, 10.1016/j.pharep.2018.08.004. 30368226

[27] S. A. Zaitone , D. M. Abo‐Elmatty , and A. A. Shaalan , “Acetyl‐l‐Carnitine and α‐Lipoic Acid Affect Rotenone‐Induced Damage in Nigral Dopaminergic Neurons of Rat Brain, Implication for Parkinson's Disease Therapy,” Pharmacology Biochemistry and Behavior 100 (2012): 347–360, 10.1016/j.pbb.2011.09.002. 21958946

[28] S. FLEMING , “Behavioral and Immunohistochemical Effects of Chronic Intravenous and Subcutaneous Infusions of Varying Doses of Rotenone,” Experimental Neurology 187 (2004): 418–429, 10.1016/j.expneurol.2004.01.023. 15144868

[29] M. M. Bradford , “A Rapid and Sensitive Method for the Quantitation of Microgram Quantities of Protein Utilizing the Principle of Protein‐Dye Binding,” Analytical Biochemistry 72 (1976): 248–254, 10.1016/0003-2697(76)90527-3. 942051

[30] H. Aebi , “Catalase,” in Methods of Enzymatic Analysis, Second Ed., ed. H. U. Bergmeyer , (Academic Press, 1974), 673–684.

[31] H. P. Misra and I. Fridovich , “The Purification and Properties of Superoxide Dismutase From *Neurospora Crassa* ,” Journal of Biological Chemistry 247 (1972): 3410–3414, 10.1016/S0021-9258(19)45155-7. 4337853

[32] H. Okhawa , N. Ohesli , and K. Yagi , “Assay of Lipid Peroxidation in Animal Tissue by Thiobarbituric Acid Reaction,” Analytical Biochemistry 95 (1979): 351–358. 36810 10.1016/0003-2697(79)90738-3

[33] J. Sedlak and R. H. Lindsay , “Estimation of Total, Protein‐Bound, and Nonprotein Sulfhydryl Groups in Tissue With Ellman's Reagent,” Analytical Biochemistry 25 (1968): 192–205, 10.1016/0003-2697(68)90092-4. 4973948

[34] J. S. Farias , K. M. Santos , N. K. S. Lima , et al., “Maternal Endotoxemia Induces Renal Collagen Deposition in Adult Offspring: Role of NADPH Oxidase/TGF‐β1/MMP‐2 Signaling Pathway,” Archives of Biochemistry and Biophysics 684 (2020): 108306, 10.1016/j.abb.2020.108306. 32081684

[35] A. Dovonou , C. Bolduc , V. Soto Linan , C. Gora , M. R. Peralta III , and M. Lévesque , “Animal Models of Parkinson's Disease: Bridging the Gap Between Disease Hallmarks and Research Questions,” Translational Neurodegeneration 12 (2023): 36, 10.1186/s40035-023-00368-8. 37468944 PMC10354932

[36] S. Ramesh and A. S. P. M. Arachchige , “Depletion of Dopamine in Parkinson's Disease and Relevant Therapeutic Options: A Review of the Literature,” AIMS Neuroscience 10 (2023): 200–231, 10.3934/Neuroscience.2023017. 37841347 PMC10567584

[37] I. Kawahata , D. I. Finkelstein , and K. Fukunaga , “Dopamine D1–D5 Receptors in Brain Nuclei: Implications for Health and Disease,” Receptors 3 (2024): 155–181.

[38] R. Viaro , F. Longo , F. Vincenzi , K. Varani , and M. Morari , “L‐DOPA Promotes Striatal Dopamine Release Through D1 Receptors and Reversal of Dopamine Transporter,” Brain Research 1768 (2021): 147583, 10.1016/j.brainres.2021.147583. 34284020

[39] F. Stocchi , D. Bravi , A. Emmi , and A. Antonini , “Parkinson Disease Therapy: Current Strategies and Future Research Priorities,” Nature Reviews Neurology 20 (2024): 695–707, 10.1038/s41582-024-01034-x. 39496848

[40] N. Malek , D. Swallow , K. A. Grosset , O. Anichtchik , M. Spillantini , and D. G. Grosset , “Alpha‐Synuclein in Peripheral Tissues and Body Fluids as a Biomarker for Parkinson's Disease—A Systematic Review,” ACTA Neurologica Scandinavica 130 (2014): 59–72, 10.1111/ane.12247. 24702516

[41] X. Dong‐Chen , C. Young , Y. Yang , S. Chen‐Yu , and P. Li‐Hua , “Signaling Pathways in Parkinson's Disease: Molecular Mechanisms and Therapeutic Interventions,” Signal Transduction and Targeted Therapy 8 (2023): 73. 36810524 10.1038/s41392-023-01353-3 PMC9944326

[42] P. J. Gaskill and H. Khoshbouei , “Dopamine and Norepinephrine are Embracing Their Immune Side and so Should We,” Current Opinion in Neurobiology 77 (2022): 102626, 10.1016/j.conb.2022.102626. 36058009 PMC10481402

[43] R. M. Ransohoff , “How Neuroinflammation Contributes to Neurodegeneration,” Science 353 (2016): 777–783, 10.1126/science.aag2590. 27540165

[44] Y. Li , N. Chen , C. Wu , et al., “Galectin‐1 Attenuates Neurodegeneration in Parkinson's Disease Model by Modulating Microglial MAPK/IκB/NFκB Axis Through its Carbohydrate‐Recognition Domain,” Brain, Behavior, and Immunity 83 (2020): 214–225, 10.1016/j.bbi.2019.10.015. 31669519

[45] Y. Nishikawa , M. E. Choudhury , K. Mikami , et al., “Anti‐Inflammatory Effects of Dopamine on Microglia and a D1 Receptor Agonist Ameliorates Neuroinflammation of the Brain in a Rat Delirium Model,” Neurochemistry International 163 (2023): 105479, 10.1016/j.neuint.2023.105479. 36608872

[46] G. Rossignoli , K. Krämer , E. Lugarà , et al., “Aromatic L‐Amino Acid Decarboxylase Deficiency: A Patient‐Derived Neuronal Model for Precision Therapies,” Brain 144 (2021): 2443–2456, 10.1093/brain/awab123. 33734312 PMC8418346

[47] J. S. Brito , A. O. Marinho , L. C. B. B. Coelho , et al., “Toxicity and Antitumor Activity of the Water‐Soluble Lectin From *Moringa oleifera* Lam. Seeds (WSMoL) in Sarcoma 180‐Bearing Mice,” Toxicon 234 (2023): 107306, 10.1016/j.toxicon.2023.107306. 37778740

[48] A. N. S. Santos , M. C. Barros , C. S. V. Souza , et al., “Safety Assessment and Hypoglycemic Effect of *Moringa oleifera* Seed Lectin (WSMoL) in Alloxan‐Induced Diabetic Mice,” Journal of Applied Toxicology (2026), 10.1002/jat.70161. PMC13628414 41847899

[49] G. R. S. Ferreira , T. L. S. Lira , and T. H. Napoleão , “Targeting Yeast Pathogens With Lectins: A Narrative Review From Mechanistic Insights to the Need for Addressing Translational Challenges,” Biomedicines 14 (2026): 105, 10.3390/biomedicines14010105. 41595640 PMC12839402

