# Supplementary material for: Moringa oleifera Lam. Seed Lectin (WSMoL) Attenuates Parkinsonian Symptoms in Mice via Anti‐Inflammatory, Antioxidant, and Neurotransmitter‐Modulating Effects
Source: Chem Biodivers. 2026 Aug 31;23(9):e71646. doi: 10.1002/cbdv.71646 (PMC13529268; doi:10.1002/cbdv.71646)
Supplement: Supplementary file 1 [file CBDV-23-e71646-s001.pdf]

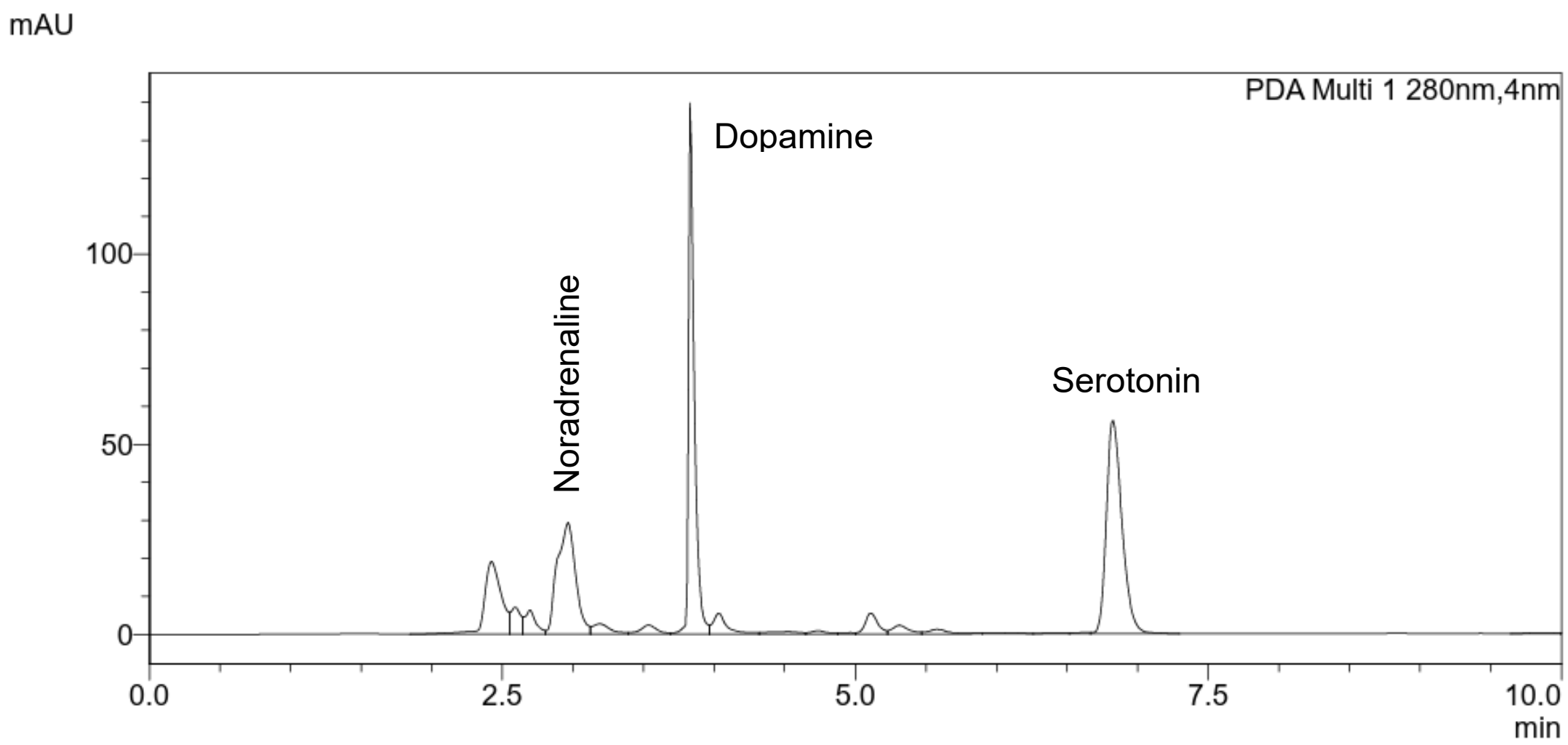

Figure S1. A representative chromatogram of the separation of noradrenaline, dopamine, and serotonin in reverse-phase mode using a sequential arrangement of Zorbax C8 (4.6 × 150 mm, 3.5 µm) and Shim-pack C18 (4.6 × 250 mm, 5 µm) analytical columns. Detection was carried out at 280 nm, with an injection volume of 50 µL and a flow rate of 1 mL/min of 0.11% phosphoric acid/acetonitrile (95:5, v/v). The displayed sample is a mixture of the three standards at 12 mg/L of each monoamine.
